# Effectiveness of the Anatomage Table Vet Prior to Cadaveric Dissection of Dog’s Thoracic Limb Muscles: A Preliminary Assessment and Student Learning Outcomes

**DOI:** 10.3390/ani16111695

**Published:** 2026-06-01

**Authors:** Ginevra Merluzzi, Elvio Lepri, Plinio Lidano, Francesca Mercati, Cecilia Dall’Aglio

**Affiliations:** 1 Department of Veterinary Medicine, University of Perugia, Via S. Costanzo, 4, 06126 Perugia, PG, Italy; ginevra.merluzzi@dottorandi.unipg.it (G.M.); francesca.mercati@unipg.it (F.M.); cecilia.dallaglio@unipg.it (C.D.); 2 Independent Researcher, 52037 Sansepolcro, AR, Italy; avbvet@gmail.com

**Keywords:** digital anatomy, innovative teaching, digital teaching, 3D anatomy, veterinary anatomy, innovative tools, myology, domestic animals

## Abstract

The Anatomage Table Vet (ATV) is a digital tool that may facilitate the understanding of complex structures and improve visuospatial skills. This study evaluated the effectiveness of the ATV as a review method prior to cadaveric dissection and collected students’ feedback. During a sport-medicine seminar, consisting of an oral presentation and a practical anatomy review session, a small sample of veterinary students voluntarily participated in this trial. A pre-test assessed baseline knowledge on muscle identification, muscle insertions and origins and biomechanics. Twenty-nine students were divided into two groups, ATV (A) and traditional lecture (C), to review canine anatomy of the caninethoracic limb and cingulum, before compiling a post-test, represented by muscle identification on five cadaveric specimens. A feedback questionnaire was administered afterwards. Mean post-test scores were significantly higher in both groups in all subsections and total score (A pre = 2.40; A post = 6.60; C pre = 2.43; C post = 6.57). No significant difference was observed in improvement between groups. Student feedback was generally positive. From the results limited to this preliminary assessment, the ATV seems well-received by students. Further research with a larger sample size is needed to clarify its educational impact.

## Simple Summary

Understanding animal anatomy is essential for veterinary students, but its study can be challenging, especially when imagining how muscles and organs are arranged in three dimensions. Traditional lectures and textbooks mainly rely on bidimensional images, which may not fully support this type of learning. New digital tools, such as interactive three-dimensional anatomy tables, have been developed to help students better visualize the animal body. This study evaluated the use of the Anatomage Table Vet as a support tool for reviewing the anatomy of the dog’s thoracic limb. Veterinary students attended a seminar on canine sports medicine and were then divided into two groups: one reviewed anatomy using the virtual table, while the other attended a traditional frontal lecture. Students were tested on muscle recognition before and after the review session, and their opinions about the learning experience were collected through a questionnaire. Results showed that all students improved their ability to recognize muscles, regardless of the teaching method used. However, students who experienced the Anatomage Table Vet reported higher interest and satisfaction. These findings suggest that three-dimensional digital tools can enhance student engagement and represent a valuable complement to traditional anatomy teaching, helping universities adapt education to modern learning styles.

## 1. Introduction

In recent years, the profile of students entering health-related disciplines has progressively shifted toward what is often described as a “digitally native” generation. These students are accustomed to interactive, visually rich, and technology-mediated forms of learning, and often express a clear preference for educational environments that promote engagement, autonomy, and immediate feedback [1].

Traditional lecture-based instruction, while still fundamental, may not fully meet these expectations, particularly in subjects that rely heavily on visualization and active exploration. As a result, universities are increasingly integrating digital resources, simulation tools, and three-dimensional visualization platforms to better align teaching strategies with contemporary learning styles and to foster a more student-centered educational experience [2]. Visuospatial ability is defined as the capacity to mentally manipulate three-dimensional structures and is widely acknowledged as an essential skill for medical and veterinary students, as well as surgical trainees. This recognition has contributed to the increasing integration of three-dimensional models and innovative educational technologies into anatomy teaching [2]. In veterinary anatomy, the ability to mentally visualize spatial relationships is critical, since students must not only recognize individual anatomical structures but also understand their position related to surrounding organs across different species. Although textbooks and anatomical atlases provide high-quality two-dimensional illustrations, they are inherently limited in their capacity to convey the complex three-dimensional relationships required for a thorough anatomical understanding of complex structures, making 3D research and the development of 3D models an important asset in veterinary anatomy education.

Modern pedagogical approaches increasingly emphasize active learning, where students engage directly with the material rather than passively receiving information. Methods such as flipped classroom, blended learning, and team-based activities encourage learners to construct knowledge through exploration, collaboration, and problem solving [3,4,5].

Following the concept of integrating multimodal didactic resources in the anatomical didactic curriculum, 3D anatomy tools offer several advantages, as they allow students to manipulate structures, perform virtual dissections, and visualize spatial relationships in a dynamic and interactive way. These tools can also facilitate group discussion, peer instruction, and inquiry-based learning, making them particularly well suited to environments where hands-on engagement and visual reasoning are central to the acquisition of anatomical competence [6].

Several studies, particularly in human medicine and dentistry, have investigated the impact of new technologies and teaching strategies on anatomy education. While many of these studies report a preference among students for three-dimensional tools over traditional methods, conclusive evidence regarding their superior efficacy remains limited [7,8]. Among the technological innovations in this field is the Anatomage Table Vet (ATV), a three-dimensional virtual dissection platform developed by Anatomage Inc. (Santa Clara, CA, USA). Initially focused on radiology and dental imaging, the company has expanded its scope to include full-body human and animal anatomical modeling using Invivo software (Invivo5, Ege University Technopark, İzmir, Turkey), which integrates CT, MRI, ultrasound, and radiographic data to generate 3D anatomical models of various animal species [9,10,11,12]. The ATV features a large interactive touchscreen (127 cm × 76 cm) that allows users to perform digital dissections and interact with highly detailed anatomical models. Individual structures can be added or removed, either separately or by layer, facilitating a clear visualization of spatial relationships and internal anatomy [12,13,14]. While the human Anatomage Table (AT) includes both male and female bodies, the veterinary version currently provides only female canine and feline models. The platform can be employed individually or in group settings and is suitable for both self-directed learning and formal instruction [10,15,16,17].

Other three-dimensional anatomy platforms currently available include EasyAnatomy, 4D Anatomy, Biosphera, and IMAIOS, each offering different technical features and educational applications. 4D Anatomy employs an interactive visualization approach comparable to that of the Anatomage Table, although its content is primarily focused on human anatomy. Biosphera provides digitally reconstructed models based mainly on theoretical anatomical representations and includes a wider range of animal species than the Anatomage Table Vet, which is currently limited to canine and feline models. IMAIOS offers both human and veterinary anatomical resources through web-based and mobile applications, facilitating flexible access across devices. EasyAnatomy has also expanded its educational tools toward augmented reality environments, providing additional immersive learning opportunities. Despite the availability of these alternatives, the Anatomage Table Vet was selected for the present study because it offers a combination of 3D and tomographic views of the muscular structures, increasing the visuospatial competence of the students, and its large interactive display and dissection-style navigation were considered particularly suitable for group teaching activities and for integration with cadaver-based practical sessions [18,19,20,21]. The human AT has also shown promise in clinical settings, including surgical planning and radiology [22,23,24,25]. To date, its use in veterinary education has been limited to a few studies largely focusing on its potential use as an innovative teaching tool [26,27]. Before integrating new tools like the ATV into existing curricula, it is essential to rigorously assess their educational value and gather feedback on student engagement and acceptance of the tool [9]. While multiple studies have explored student experiences with the human AT, often reporting positive impressions with occasional technical limitations [1,10,14,28], the veterinary version remains largely untested in this context. The growing interest in new technologies is driving current research, with the aim of evaluating novel tools as teaching resources and determining whether they should complement or substitute existing teaching strategies. At present, evidence is insufficient to justify the elimination of cadaveric dissection in veterinary and cadaveric dissection remains the gold standard. However, for economic, ethical, and practical reasons, the number of cadavers available for dissection is constantly decreasing, and technologies such as the ATV may help mitigate challenges related to cadaver availability by providing both instructors and students with an alternative to traditional dissection practice [29].

This study was associated with a sports medicine seminar organized at the University of Perugia, with a focus on the most common thoracic limb lesions occurring in agility dogs. Since the seminar required a review of the anatomical structures relevant to the topic, to allow students to more easily understand the biomechanical and diagnostic imaging correlations, it provided an ideal opportunity to test the ATV as an anatomical revision tool.

Given that participating students were in the final two years of the veterinary program and had already passed their anatomy examination, the ATV was used specifically as a tool for reviewing anatomical notions they were expected to have previously acquired. Accordingly, the aims of this study were twofold: first, to assess the effectiveness of the ATV as a revision tool prior to cadaveric dissection, and second, to gather student feedback on its usability.

## 2. Participants, Materials and Methods

### 2.1. Participants

A total of 29 students voluntarily participated in the study and were randomly assigned to one of two groups: the Anatomage group (Group A, n = 15) and the control group (Group C, n = 14). Only students enrolled in the last two years of the Veterinary Medicine course were included in this study and the successful undertaking of the veterinary anatomy exam was set as the inclusion criteria.

### 2.2. Materials

The study project was approved by the Bioethics Committee of the University of Perugia (Prot. Non. 314952). An Informed Consent Document, detailing the study procedures, was provided to the students before activities begin. To ensure privacy, participants used a five-digit alphanumeric identification code instead of providing their name when compiling the pre-test, post-test and feedback questionnaire that were used in this activity.

### 2.3. Study Design

As part of the outgoing orientation program organized at the University of Perugia, a seminar on canine sports medicine focusing on the most common lesions affecting the thoracic limb in agility dog, was held for veterinary students.

In parallel with the seminar, a brief pilot study was organized to evaluate the use of the ATV as an educational tool.

The decision to combine the present study with a seminar on sports medicine was driven by the intention to promote the use of emerging educational technologies beyond the formal curricular setting, encouraging their integration across the broader range of academic activities within the university.

Sports medicine was selected as the seminar topic as it represents a field that lies at the intersection between the standard veterinary curriculum and professional practice. The seminar integrated clinical concepts with diagnostic imaging and the use of the anatomical table, thereby providing a multidisciplinary and applied context for the educational intervention.

The muscular structures included in the trial were selected based on their clinical relevance in agility dogs. Consequently, the focus was placed on the shoulder and brachial musculature, which are commonly involved in overuse and acute traumatic conditions in canine athletes. These muscles are generally larger and easier to identify compared with smaller and more intricate muscles of the canine antebrachium. However, the latter were deemed less suitable for inclusion in the present study, as they were not directly aligned with the thematic and clinical objectives of the seminar. The ATV (v11) was used as the designated tool for anatomical revision during the training session of the experimental group. A bidimensional presentation was prepared for the training session of the control group.

#### 2.3.1. Pre-Test

**Figure 1:**
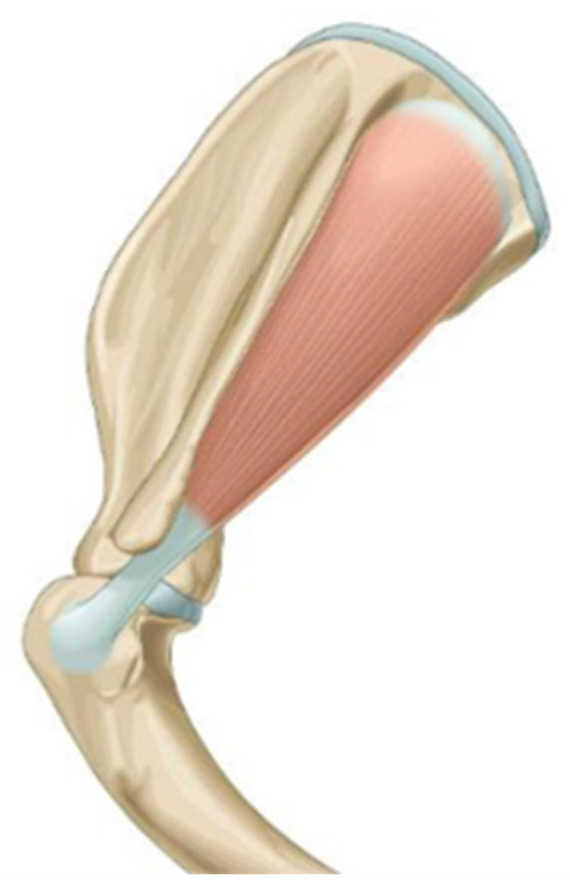
Example of pictures used in the pre-test for muscle recognition [31].

Prior to the seminar, all participants completed a 15-min pre-test administered simultaneously to the whole group. The pre-test consisted of ten questions aimed at assessing baseline knowledge of canine thoracic limb anatomy and function. Specifically, five items required the identification of selected muscles using photographic images (Figure 1), two items queried muscle origins and insertions or distinctive osteological features of the region, and three items focused on biomechanical aspects and clinical concepts later addressed in the seminar.

This assessment provided a standardized baseline for evaluating students’ knowledge before any intervention [30].

#### 2.3.2. Seminar and Revision Session

Following the pre-test, all students attended a 2-h seminar on canine sports medicine. The seminar did not include any anatomical content relevant to the subsequent anatomical review session. Immediately after the seminar, participants were randomly allocated into two groups for a 30-min anatomy review session designed to reinforce the structures and functional concepts relevant to the thoracic limb. Both review sessions were delivered by the same instructor, following a fixed sequence of topics to minimize confounding factors, and leave the instructional modality as the only difference between groups.

Students from group A received the revision session using the ATV. The instructor manipulated the three-dimensional digital canine model in real time to highlight the muscles of interest, their origins and insertions, spatial relationships, and biomechanical functions. When relevant, brief clinical reminders were integrated to connect the anatomical concepts to issues discussed during the seminar.

Group C students attended a traditional frontal lecture covering the same content. Visual support consisted of two-dimensional slides and images extracted from reference books. No interactive or three-dimensional tools were used.

#### 2.3.3. Post-Test and Feedback Questionnaire

Immediately after the review session, students received the post-test and were individually directed to the necropsy room for a practical assessment [30]. The post-test mirrored the structure of the pre-test, again consisting of ten questions: five on muscle recognition, two on origins/insertions or bone landmarks, and three on biomechanics and clinical concepts. The key difference was that muscle recognition was now assessed on cadaveric specimens rather than photographic images. Five dissection tables were prepared, each displaying a canine cadaver with a single muscle identified using pins or markers. The cadavers were collected on the weeks leading up to the seminar and had been skinned and frozen. Only the necessary body portions had been conserved. Muscles that required superficial structures to be removed for easier access were already uncovered to facilitate recognition. Each student was allowed 10 min to rotate sequentially through the stations, with approximately two minutes per table, and were free to allocate more time to a specific table if recognition of that muscle proved more difficult, provided that the overall time limit was not exceeded. At the end of the rotation, an additional five minutes were provided to complete the remaining theoretical questions on the post-test form. Students were not allowed to access the necropsy room at the same time and a supervisor was present throughout the activity to avoid exchanges of information between participants.

The muscles included across the pre- and post-test assessments comprised the trapezius, serratus ventralis, brachialis, biceps brachii, teres major, teres minor, and deltoid muscles. All questions present in the post-test were different than the ones included in the pre-test.

As part of the post-test, all participants also filled out a feedback questionnaire consisting of six statements rated on a 5-point Likert scale (1 = strongly disagree; 5 = strongly agree) [32]. The questionnaire explored students’ perceptions of the overall trial, including clarity, usability and didactic value of the teaching method experienced, as well as their general satisfaction with the learning activity (Table 1).

**Figure 2:**
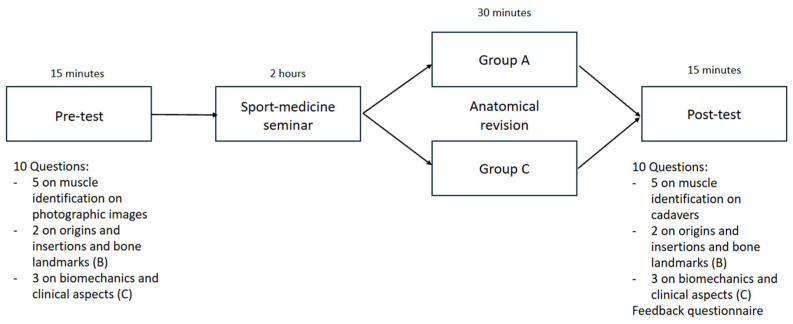
Flow diagram of participant progression through the study.

A diagram depicting the progression of the participants through the study activities is shown in Figure 2.

### 2.4. Statistical Analysis

For the purpose of this study, all 10 items present in the pre- and post-tests were scored and included in the statistical analysis, and sub-scores were calculated.

Data were analyzed using commercially available statistical software (JASP version 0.16.1; The JASP Team, University of Amsterdam, Amsterdam, The Netherlands). A descriptive statistic was used for answers to the affirmations presented in the feedback questionnaire and pre-test and post-test scores. Mann–Whitney U tests were conducted to test for significant differences in pre-test scores and questionnaire answers between groups. Wilcoxon signed-rank test was used to evaluate within-group improvement from pre- to post-test for each subscore (muscle recognition, insertions and bone landmarks, biomechanics and clinical). A mixed ANOVA test was used to examine the effect of time (pre- vs. post- test) and group (A vs. C) on students’ performance in each sub-score. Significance was set at *p* < 0.05.

## 3. Results

### 3.1. Feedback Questionnaire

Descriptive statistics for the responses to the satisfaction questionnaire are presented in Table 2. Overall, students in group A reported slightly higher scores regarding the perceived usefulness of and interest in the ATV (Q4, Q6).

Descriptive statistics of responses provided by the Anatomage (Group A) and Control (Group C) groups to six statements (Q1–6) in the feedback questionnaire. Responses were scored on a 5-point Likert scale (1 = strongly disagree; 5 = strongly agree). Between-group comparisons were performed using the Mann–Whitney U test, and corresponding *p*-values are reported for each statement. A statistically significant difference (*p* < 0.05) was observed for Q3. Although most questionnaire items did not show statistically significant differences between the two groups, one notable exception emerged for Q3. For this statement, group C exhibited a significantly higher level of agreement (*U* = 41.50, *p* = 0.005).

### 3.2. Quiz Scores

In the pre-test, both groups (A and C) showed score distributions mainly concentrated at the lower end of the scale. In group A, scores were evenly distributed across values 0, 1, and 2, with fewer observations at 3. Group C displayed a more pronounced clustering at score 1, while higher scores were less frequent. Although the mean score for all subsections in the pre-test was slightly different in the two groups, this difference was not statistically significant. This indicates that the two groups were comparable in their baseline performance, supporting the assumption of similar prior anatomical knowledge before the intervention.

In the post-test, both groups achieved higher scores in all subsections and total score.

Both groups demonstrated substantial gains in the post-test, with Wilcoxon analyses confirming significant within-group improvement (*p* < 0.01). However, the magnitude of this improvement did not differ significantly between the two instructional methods, as the mixed ANOVA analysis did not observe a significant interaction between group and time, suggesting that both groups benefited similarly from their respective review sessions. Distribution of scores achieved by both groups in the pre and post-test are shown in Table 3.

## 4. Discussion

The present study sought to evaluate the effectiveness of the ATV as a myology revision tool before entering the necropsy room and collect student feedback on the use of the instrument in a seminar context. This preliminary assessment was conducted independently of curricular teaching activities to avoid any potential interference with students’ academic performance and to prevent possible disparities in the educational offer.

### 4.1. ATV Effectiveness

This study’s findings showed a significant improvement in performance from pre- to post-test in both groups (A and C), yet no significant differences in the entity of improvement emerged between them. This indicates that, although the revision session was beneficial overall, the use of the ATV under the specific conditions of this trial did not yield measurably superior short-term outcomes compared to conventional teaching.

This improvement across both groups is unsurprising, as the anatomical revision session was delivered immediately prior to the post-test, giving all participants a targeted and focused training session shortly before the activity in the necropsy room.

The similarity in results achieved by both groups could be explained by the instructional modality adopted. In our protocol, the ATV was operated exclusively by the instructor, while students were not given the opportunity to interact directly with the 3D interface. This passive mode of use may have reduced the educational advantage of the ATV comparingto that of the control method, which relied on static 2D images.

Overall, the comparable findings between Group A and Group C test scores are in accordance with the broader literature, which shows mixed evidence on the human AT’s impact on students’ performance [13,33,34,35,36,37]. While some studies have reported improved outcomes following virtual dissections compared with cadaver-based teaching [38], others have found no significant differences in learning achievement when comparing AT-assisted instruction with conventional cadaveric approaches for musculoskeletal anatomy [39].

### 4.2. Students’ Feedback

Analysis of the questionnaire responses revealed that students in Group A showed higher levels of satisfaction with the teaching tool, a finding likely influenced by their direct exposure to the technology. Conversely, several students in Group C expressed a desire for a different revision method, as indicated by their significantly greater agreement with Q3. This is plausibly tied to their awareness of not having been assigned to the ‘experimental’ group and their interest in experiencing the ATV for the same activity. Students across both groups expressed enthusiasm for participating in future activities involving the ATV and provided positive feedback regarding its potential value as a revision tool for veterinary anatomy.

These observations align with previous reports showing that, regardless of variable evidence on learning outcomes, students generally perceive the human AT as highly engaging and helpful for visualizing topographic relationships [40]. In many cases, learners have also preferred it over traditional textbook-based study and have reported heightened engagement during sessions in which the human AT was used [41,42,43]. Notably, student acceptance and appreciation of the human AT tend to increase with repeated exposure, suggesting that successful curricular integration depends not only on the implementation of innovative technologies but also on sufficient user familiarity and training [5].

The limited familiarity of the students involved in this study with the ATV has been reflected by the consistently low scores for item Q5 on the questionnaire. Allowing untrained students to manipulate the tool during the training session risked compromising the learning experience, thereby justifying the instructor-led approach.

### 4.3. Future Prospects

The study was designed within the context of a broader series of small-scale investigations aimed at evaluating the effectiveness of the ATV and the optimal strategies for its curricular implementation at the University of Perugia. A previous study focused on the use of the ATV for theoretical anatomy teaching, specifically the Triadan dental classification system, which requires minimal visuospatial reasoning [44]. The present study expands on that work by examining a more visuospatially demanding anatomical subject, namely the muscles of the canine thoracic limb and thoracic limb cingulum. A logical next step will be to explore the relative benefits of active versus passive engagement with the ATV. Future research should recruit larger cohorts, incorporate longitudinal follow-up assessments, and implement study designs that permit active student engagement with the ATV.

### 4.4. Limitations

This study presents limitations that should be acknowledged. Firstly, the sample size was relatively small, which may limit the generalisability of the findings and reduce statistical power to detect subtle differences between groups. Second, the ATV was used in a passive, instructor-led mode, potentially underestimating its pedagogical potential. Finally, performance was assessed using a single post-intervention test, which does not allow for evaluation of long-term retention or the durability of learning gains.

## 5. Conclusions

Based on the findings of this study, both traditional and ATV-assisted teaching methods seem to significantly improve students’ muscle recognition skills when used as revision tools before cadaveric dissection. However, this preliminary assessment’s findings suggest that passive exposure to 3D digital tools may not provide substantial added value over conventional approaches. Future research should explore the benefits of active versus passive use of such tools and aim to identify effective strategies for their integration into veterinary curricula. Additionally, a larger cohort of students should be involved, as the sample of this study was relatively small. Promoting early student familiarity and competence with innovative technologies could enhance both the usability and educational impact of these new tools.

## Figures and Tables

**Table 1:** Feedback questionnaire handed to the students at the end of the trial. The students answered each statement using a five-point Likert scale based on how much they related to the individual affirmation (1 = strongly disagree; 2 = disagree; 3 = neutral opinion; 4 = agree; 5 = strongly agree) [32].

Q1	I was familiar with the anatomical regions discussed during the seminar.
Q2	The training session before the cadaveric practical test was useful for reviewing the anatomy of the thoracic limb cingulumand thoracic limb
Q3	I would have preferred a different method to review the anatomy before the cadaveric test.
Q4	I am interested in participating in additional activities involving the Anatomage Table Vet.
Q5	I was already familiar with the Anatomage Table Vet.
Q6	I consider the Anatomage Table Vet a useful tool for reviewing veterinary anatomy.

**Table 2:** Student feedback on the learning experience.

Question	Group	Mean ± SD	*p*-Value
Q1	A	3.00 ± 1.42	0.141
	C	2.21 ± 0.80	
Q2	A	4.53 ± 0.64	0.418
	C	4.36 ± 0.63	
Q3	A	1.80 ± 1.15	0.005 *
	C	3.50 ± 1.51	
Q4	A	4.40 ± 0.83	0.299
	C	4.07 ± 1.21	
Q5	A	2.27 ± 1.58	0.620
	C	1.79 ± 0.98	
Q6	A	4.60 ± 0.74	0.669
	C	4.50 ± 0.76	

* *p*-value < 0.05 indicates a statistically significant difference in answers to Q3 between the two groups.

**Table 3:** Pre- and post-test scores (mean ± SD) for muscle identification, bone landmarks, and biomechanics/clinical applications in the Anatomage (A) and Control (C) groups. Within-group changes were assessed with Wilcoxon signed-rank tests, between-group differences over time with mixed ANOVA, and baseline differences with Mann–Whitney tests.

Test	Group	Pre-Test (Mean ± SD)	Post-Test (Mean ± SD)	*p* (Wilcoxon)	*p* (Time × Group—Mixed ANOVA)	*p* Baseline (Mann–Whitney)
Muscle identification	A	1.20 ± 1.08	3.40 ± 1.50	<0.01 *	0.493	0.818
	C	1.07 ± 0.83	3.64 ± 0.93	<0.01 *		
Bone landmarks, origins and insertions	A	0.47 ± 0.52	1.13 ± 0.52	<0.01 *	0.949	0.980
	C	0.57 ± 0.85	1.21 ± 0.89	<0.01 *		
Biomechanics/clinical applications	A	0.93 ± 0.96	2.13 ± 0.74	<0.01 *	0.356	0.235
	C	0.93 ± 0.83	1.79 ± 0.80	<0.01 *		
Total Score	A	2.40 ± 1.81	6.60 ± 1.72	<0.01 *	0.939	0.841
	C	2.43 ± 1.65	6.57 ± 1.56	<0.01 *		

* *p*-value < 0.05 indicates a statistically significant difference in post-test scores compared to pre-test scores in all categories.

## Data Availability

The original contributions presented in this study are included in this article. Further inquiries can be directed to the corresponding author.

## Abbreviations

The following abbreviations are used in this manuscript:

ATV	Anatomage Table Vet
AT	Anatomage Table
